# Minimally Invasive Robotic-Assisted Cystolithotomy in a Complicated Urinary Diversion: A Feasible and Safe Approach

**DOI:** 10.1155/2021/8345092

**Published:** 2021-12-14

**Authors:** A. Haffar, C. Crigger, T. Trump, M. Jessop, M. W. Salkini

**Affiliations:** ^1^West Virginia University School of Medicine, West Virginia, USA; ^2^Johns Hopkins Children's Center, Baltimore, USA; ^3^Department of Urology, West Virginia University, West Virginia, USA; ^4^Benefis Health System Foundation, Great Falls, USA

## Abstract

Urinary diversion following radical cystectomy and neoadjuvant chemotherapy is the gold standard for the management of muscle-invasive bladder cancer. Urinary diversions are at an increased risk of urolithiasis as a result of various factors. Traditional surgical intervention has included open cystolithotomy which has given way to minimally invasive techniques as of late. We describe a case of a robotic-assisted cystolithotomy from a neobladder in a 54-year-old female patient with muscle-invasive bladder cancer. This is the first description of a robotic-assisted removal of a stone in an orthotopic neobladder. This approach has many advantages, especially in the removal of larger stones. Further study is needed to investigate the efficacy and success of this approach.

## 1. Introduction

The standard of care for muscle-invasive bladder cancer is neoadjuvant chemotherapy followed by radical cystectomy with bilateral pelvic lymph node dissection [1]. Urologists must discuss types of urinary diversions in detail preoperatively to maximize patient understanding and manage patient expectation postoperatively. Though different in their technique, ileal conduits and orthotopic neobladders allow patients to recover their normal lifestyle after a cystectomy. Patients with neobladders often report better self-image and a more active lifestyle [2]. Despite the benefits of these urinary diversions, they can give rise to multiple complications. Urolithiasis is a known complication of urinary diversions, and there are many factors that can influence this process. The use of intestinal segments to create a urinary diversion can cause urolithiasis from continued bicarbonate loss in the urine, hyperoxaluria, urinary stasis, excessive mucus production, and the use of surgical staples in the construction of the neobladder [3, 4].

In patients who develop urolithiasis, surgical management has traditionally involved open cystolithotomy, transurethral, and percutaneous approaches. While the merits and drawbacks of each are open to debate, a delicate balance exists—minimizing treatments to ensure a stone-free rate without compromising the reconstructed bladder. Despite improvements in robotics, a robotic approach has not been widely adopted. In what follows, we describe a robotic approach for the removal of calculus from a reconstructed bladder.

## 2. Case Presentation

**Figure 1:**
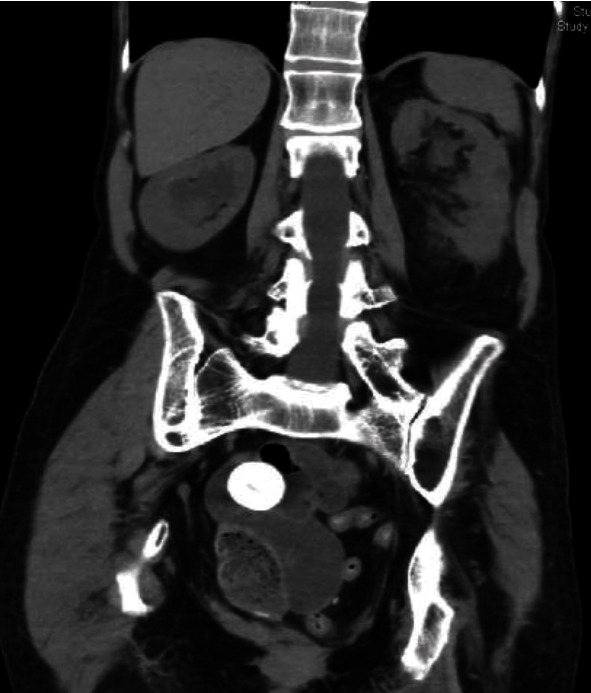
Computed tomography (coronal view) of abdomen and pelvis revealing the 3.1 cm calculus in question.

A 54-year-old female was referred to an academic tertiary center's urology clinic for evaluation of bilateral hydronephrosis, recurrent urinary tract infections complicated by multiple instances of sepsis, a right ureteropelvic junction stone, and left renal abscess. Her urologic history is complicated by a history of muscle-invasive bladder cancer from 23 years prior status-post open cystectomy with subsequent undiversion without removal of neobladder. Her cystectomy and subsequent undiversion were both done at an outside institution, and the reason for not removing the neobladder at that time is unknown. She also has a history of tethered cord syndrome and neurogenic bowel with a colostomy. Bilateral nephrostomy tubes were placed, and she was treated for her abscess. After stabilizing, she underwent bilateral anterograde nephrostograms followed by bilateral antegrade ureteroscopy which revealed that her ureteroenteric anastomoses were patent bilaterally. Her ureteropelvic junction calculus was removed at this time. Imaging also demonstrated a 3.1 cm stone in her original neobladder which the patient elected to have removed (Figure 1). Her prior urologic infections were treated with outpatient IV Rocephin for a total of 6 weeks

The patient was taken to the operative suite for planned robot-assisted laparoscopic cystolithotomy with stone extraction. Under general anesthesia, the patient was placed in a dorsal lithotomy position. Preoperative cefazolin was administered. A Foley catheter was introduced into the conduit. A midline incision was made above the umbilicus, dissecting down to the peritoneum, and a 5/12 mm trocar was introduced via the Hassan technique. Two 8 mm trocars were placed 8 cm to either side of the camera trocar incision. A 5/12 mm assistant trocar was added lateral to the right robotic arm trocar. The patient was then placed in a steep Trendelenburg position, and the robot was docked. Multiple adhesions of the mesentery to the abdominal wall were lysed, allowing the colostomy and conduit to be easily identified. Initially, we believed the stone to be in the neobladder within the pelvis, so this was opened with monopolar cautery parallel to the blood supply, but the stone was not able to be visualized. We were then able to find the stone in the neobladder deeper in the pelvis, visible through the wall of the diversion. This area was opened, and the stone was able to be fully visualized. At the time of extraction, the stone was fractured inadvertently, likely due to the softer nature of the stone, requiring it to be removed in two pieces. The pieces were removed with the aid of an EndoCatch bag without the need to extend any fascial incisions. The neobladder was then closed in two layers using 3-0 Vicryl V-loc sutures, and a 15 Fr Blake drain was placed alongside the conduit. The catheter was then removed, and the trocar incisions were closed, terminating the procedure. Estimated blood loss was 50 mL. On admission, the patient's BUN was 31, and on the first postoperative day, it was 36. The patient tolerated the procedure well, and the postoperative course was uneventful with discharge occurring on the first postoperative day. JP creatinine on the first postoperative day was negative, and the patient was discharged the same day. She was discharged on DS bactrim BID for 10 days. The drain remained in place at discharge due to higher output and for monitoring of the closure. The output remained less than 50 cc/day per patient documentation and was removed without incident at her 2-week postoperative follow-up, where the patient denied any complaints. She reported consistent urine output from her urostomy with occasional mucous. The patient visited the ED approximately 5 weeks postop with concerns for constipation from her colostomy and at that point was found to have urinalysis concerning for UTI and started on Levaquin for 7 days. CT imaging from 4 weeks postop showed a 3.1 × 2.5 cm calculus within the neobladder lumen along with mild bilateral hydronephrosis similar to her preoperative imaging.

## 3. Discussion

Urolithiasis of urinary diversions is a rare complication with variable incidence rates depending on the type of diversion—13.4% of patients with ileal conduits were found to have an upper tract stone, while only 4.5% developed conduit stones [5]. However, only 3-9% of patients with an orthotopic neobladder using the Paduan technique form stones [3]. Studer et al. reported no neobladder stones in 482 patients when followed for 20 years [6]. Yet, the Kock pouch technique was found to have an incidence of up to 43%; this illustrates the importance that urinary diversion construction technique can have on complication rates [3]. As evidenced by the quick production of a new calculus, preventing stone recurrence is crucial when treating patients with diversions. Prevention relies on enhanced pouch drainage and correcting underlying metabolic abnormalities like acidosis, hyperoxaluria, and hypocitraturia [5].

Treatment of diversion stones typically involves endoscopic procedures as this is currently considered the safest method compared to open surgical procedures. However, a large stone will require extensive manipulation in the urethra, possibly jeopardizing the sphincter [7]. For this reason, others have described great success with endoscopic percutaneous cystolithotomy and adopted this approach as their preferred technique [8].

Regardless of the surgical approach utilized, the balance between optimizing stone-free rates without compromising the reconstructed bladder and limiting surgical procedures is a pressing consideration for both the patient and physician. This first description of a robotic-assisted approach for stone removal in a reconstructed bladder highlights the benefits of this technique. In addition to the many well-known benefits inherent to robotics such as 3-dimensional visualization, greater degrees of freedom in hand movements, accelerated recovery, and lower complication rates, the robotic approach specifically enabled success in our case. This patient had prior abdominal surgeries as outlined including a cystectomy with neobladder and subsequent undiversion to an ileal conduit. Given the complexity of the patient's anatomy and prior abdominal surgical history, we offered a minimally invasive approach in order to prevent an additional laparotomy incision and possible prolonged recovery associated with this approach. Obviously, this was approached with the understanding that as with any minimally invasive approach there is a chance of converting to open if the procedure cannot be safely carried out. Fortunately, this procedure was able to be conducted robotically. We believe that the main advantage of this approach was that the patient was able to be discharged on postoperative day 1 and avoided a prolonged hospital course that is typically associated with open incisions. We felt that using other approaches would not be ideal due to the high likelihood of requiring multiple procedures to address the large stone burden and difficult anatomy. The superior visualization and maneuverability afforded to us with this technique were superior to other minimally invasive techniques. While endoscopic percutaneous and transurethral procedures are typically indicated for smaller bladder stones, open cystolithotomies are reserved for larger stones [9]. However, open cystolithotomies are also associated with longer recovery times, greater morbidity, and more rapid recurrence of bladder stones [10]. This case is an excellent illustration of the role robotic-assisted procedures may play in the removal of a urinary diversion stone.

In summary, robotic-assisted cystolithotomy is a modern approach to an old problem—allowing confident removal of calculi from reconstructed bladders that may be preferable in patients with history of prior surgery or complicated anatomy. We believe that in the setting of large stones that would be difficult to remove efficiently through a percutaneous approach, a robotic approach could be more advantageous. Particularly, we believe that stone removal through a minimally invasive approach, compared to the traditional open approach in the setting of prior abdominal surgeries, benefits the patient with faster recovery, potentially less blood loss, and likely shorter duration of hospital stay. Despite the advantages of minimally invasive endoscopic procedures, such techniques may prove difficult in altered anatomy as well as when removing larger stone burdens. Further evaluation is required to evaluate the place that this robotic approach has in challenging stone removal.
